# Explore the effects of overweight and smoking on spontaneous brain activity: Independent and reverse

**DOI:** 10.3389/fnins.2022.944768

**Published:** 2022-10-12

**Authors:** Xinyu Gao, Mengzhe Zhang, Zhengui Yang, Xiaoyu Niu, Jingli Chen, Bingqian Zhou, Weijian Wang, Yarui Wei, Jingliang Cheng, Shaoqiang Han, Yong Zhang

**Affiliations:** ^1^Department of Magnetic Resonance Imaging, The First Affiliated Hospital of Zhengzhou University, Zhengzhou, China; ^2^Key Laboratory for Functional Magnetic Resonance Imaging and Molecular Imaging of Henan Province, Zhengzhou, China; ^3^Engineering Technology Research Center for Detection and Application of Brain Function of Henan Province, Zhengzhou, China; ^4^Engineering Research Center of Medical Imaging Intelligent Diagnosis and Treatment of Henan Province, Zhengzhou, China; ^5^Key Laboratory of Magnetic Resonance and Brain Function of Henan Province, Zhengzhou, China; ^6^Key Laboratory of Brain Function and Cognitive Magnetic Resonance Imaging of Zhengzhou, Zhengzhou, China; ^7^Key Laboratory of Imaging Intelligence Research Medicine of Henan Province, Zhengzhou, China

**Keywords:** amplitude of low-frequency fluctuation, nicotine addiction, smoke, overweight, body mass index

## Abstract

Accumulating evidence suggested that overweight and smoking often co-exist. However, current neuroimaging researches have almost always studied smoking or overweight status separately. Here we sought to investigate the neurobiological mechanisms of this comorbid association, by detecting spontaneous brain activity changes associated with smoking and weight status separately and collectively. We used 2 × 2 factorial design and included the following four groups: overweight/normal-weight smokers (*n* = 34/*n* = 30) and overweight/normal-weight non-smokers (*n* = 22/*n* = 24). The spontaneous brain activity among the four groups was comparable using an amplitude of low-frequency fluctuation (ALFF) method based on resting-state fMRI (rs-fMRI). Furthermore, correlation analyses between brain activity changes, smoking severity and BMI values were performed. A main effect of smoking was discovered in the default mode network (DMN) and visual network related brain regions. Moreover, overweight people had high ALFF value in the brain regions associated with reward and executive control. More importantly, smoking and overweight both affected brain activity of the middle temporal gyrus (MTG), but the effect was opposite. And the brain activity of MTG was negatively correlated with smoking years, pack year and BMI value. These results suggest that smoking and overweight not only affect spontaneous brain activity alone, but also paradoxically affect spontaneous brain activity in the MTG. This suggests that we need to control for weight as a variable when studying spontaneous brain activity in smokers. Besides, this interaction may provide a neurological explanation for the comorbidity of overweight and smoking and a target for the treatment of comorbid populations.

## Introduction

The combination of cigarette smoking and overweight in individuals can synergistically increase risk of mortality (Freedman et al., 2006). Smoking and overweight are major causes for many serious diseases worldwide (Danaei et al., 2005), and in the United States overweight was the second leading cause of preventable, premature death after smoking (Mokdad et al., 2004). And about 30 percent of obese people smoke, compared with 15 percent of the general population in the United States (Hales et al., 2018; Creamer et al., 2019). Many people believe that quitting smoking will lead to weight gain, that’s why a lot of people don’t want to abandon. Part of the crowd even chooses to relapse because of weight gain (Audrain-McGovern and Benowitz, 2011). Interventions targeting smoking and overweight independently have modest success at best (Spring et al., 2009; Aubin et al., 2012), therefore, understanding the relationship between these two conditions is more conducive to targeted treatment and curb their profound negative impact.

Many studies have been done on the relationship between smoking and weight, with mixed and even contradictory results. For example, epidemiological investigation found that current smokers tend to be leaner than never or former smokers (Plurphanswat and Rodu, 2014). And past investigations have found that when smokers stop smoking, they gain weight and are even fatter than before they started (Audrain-McGovern and Benowitz, 2011). However, a research (Kim et al., 2012) showed that smokers had a higher rate of central adiposity even if smokers are leaner than non-smokers. In addition, Chiolero et al. (2007) found that the more cigarettes a smoker smokes per day, the higher his body mass index (BMI). Mechanically, nicotine is a sympathomimetic agent that can increase adipose tissue thermogenesis by increasing lipolysis and the subsequent recycling of fatty acids into triglycerides (Hellerstein et al., 1994; Andersson and Arner, 2001). In a word, nicotine increases energy consumption via action on peripheral tissue and through regulation of brain metabolism. Smoking increases 24 h energy expenditure by ∼10% (Hofstetter et al., 1986).

Moreover, some studies (Thorgeirsson et al., 2013; Taylor et al., 2019; Wills and Hopfer, 2019; Ely et al., 2021) have indicated that there is a relationship between smoking and weight, with advances in neuroimaging. Structurally, long-term smokers showed a gray matter (GM) volume decrease in the bilateral prefrontal cortex and left insular and GM volume increase in the right lingual cortex and left occipital cortex (Yang et al., 2020). The frontal temporal cortex (including bilateral middle temporal gyrus (MTG), left insula, left precuneus, bilateral orbitofrontal cortex, and so on) was thinner in people with a high BMI (Opel et al., 2021). Functionally, an index called amplitude of low-frequency fluctuation (ALFF) reflects the spontaneous activity of neurons in the resting state by measuring the intensity of the spontaneous fluctuation area (Rubin et al., 2018; Deng et al., 2022). Particularly, previous studies demonstrated that static ALFF changes were mainly concentrated in the right inferior frontal gyrus, left middle frontal gyrus, bilateral precuneus in smokers (Liu et al., 2018; Wen et al., 2021). Also, a weight-related functional MRI study discovered that overweight people showed decreased ALFF in the right superior temporal gyrus and increased ALFF in left inferior temporal gyrus (ITG), hippocampus/parahippocampal gyrus, fusiform gyrus/amygdala and bilateral caudate (Ren et al., 2020; Zhang et al., 2020). Smokers are not a small percentage of overweight people. In conclusion, above evidence suggests that there is an overlap of brain regions that change when people smoke or are overweight are studied separately. However, despite epidemiological and some animal studies (Thorgeirsson et al., 2013; Besson et al., 2019; Wills and Hopfer, 2019), no neuroimaging studies have explored whether there is a brain interaction between smokers and overweight people by using the ALFF method.

To cover these gaps, we divided our subjects into four groups (overweight smokers/non-smokers and normal weight smokers/non-smokers) based on ALFF method. According to previous studies, we hypothesized that the interaction between smoking and weight would alter spontaneous brain activity in certain brain regions (whether in the same direction or in the opposite direction cannot be determined), and that analysis would support that overweight is an influencing factor for the brain function of smoking people. Furthermore, we also assessed the effects of smoking and being overweight on spontaneous brain activity.

## Materials and methods

### Participants

Sixty-four smokers and forty-six non-smokers were recruited and divided into four groups, including: (i) overweight smokers (*n* = 34); (ii) normal-weight smokers (*n* = 30); (iii) overweight non-smokers (*n* = 22), and (iv) normal-weight non-smokers (*n* = 24) in this study.

All subjects were right-handed males. The nicotine dependence severity was measured by Fagerström Test for Nicotine Dependence (FTND, for current effects) (Heatherton et al., 1991) and pack-year (for chronic effects, be defined as smoking years × number of cigarettes smoked per day/20). Smokers were included as follows: (1) smoking at least 1 daily for > 2 years; (2) met the DSM-IV criteria for nicotine dependence (Wu et al., 2015; Sadeghi-Ardekani et al., 2018). Sex- and age-matched non-smokers (*N* = 46) who did not currently smoke and had no history of consumption of cigarettes or any nicotine products (Liu et al., 2018) take part in this study. BMI is calculated by height (m) divided by weight squared (kg). Normal-weight participants were defined as those with a BMI less than 25.0, and overweight participants had a BMI of more than 25.0 and less than 30.0 (Ely et al., 2021).

Exclusion criteria for all groups are as follows: (1) any physical or neuropsychiatric disease; (2) urine test and self-report indicating other substance or drug abuse (except nicotine); (3) contraindications to magnetic resonance imaging.

This study was reviewed and approved by the Ethics Committee of the First Affiliated Hospital of Zhengzhou University. All examinations were conducted under the guidance of the 1975 Declaration of Helsinki (Shephard, 1976). All patients were recruited via the Internet or through advertising at the First Affiliated Hospital of Zhengzhou University. Written informed consent was obtained from all subjects.

### Imaging acquisition measures

All images were acquired on a 3.0T MRI scanner (Siemens Skyra) at the First Affiliated Hospital of Zhengzhou University. Smokers were required to smoke a cigarette 20 min prior to scanning to avoid nicotine withdrawal-related imaging after-effects. All participants were offered a snack before scanning. The participants remained stationary during the scan. All participants were requested to keep their eyes closed and relax their minds without falling asleep. Foam padding and earplugs were used to reduce subjects’ head movements and scanner noises. At the end of scanning, participants were also asked if they had fallen asleep during scanning. BOLD resting-state fMRI (rs-fMRI) images were collected using an echo-planar imaging sequence (repetition time = 2,000 ms, echo time = 30 ms, flip angle = 80°). 36 transverse slices (field of view = 240 × 240 mm^2^, matrix = 64 × 64, slice thickness = 4 mm, 180 volumes) that aligned along the AC-PC line were acquired with a total scan time of 360 s.

### Imaging analysis procedure

The rs-fMRI data were preprocessed using the Toolkit (DPARSF, V4.3, advanced edition)^1^ in MATLAB. The first 5 volumes were excluded then slice-timing and realignment. Subjects with a maximum head motion > 2.5 mm or > 2.5° head rotation were excluded. No subjects were excluded in this step. The functional images were spatially normalized and re-sampled to 3 × 3 × 3 mm^3^. Next, 24 motion parameters, global signals, cerebrospinal fluid signals and white matter signals were regressed using multiple linear regression analysis. Frame wise displacement (FD) was calculated for each time point (Power et al., 2012; Han et al., 2020), and mean FD > 0.5 mm was excluded. Functional images were spatially smoothed with a 6 mm full width at half maximum Gaussian kernel and then detrended.

ALFF was calculated in REST (rs-fMRI Data Analysis Toolkit). Fast Fourier Transform (FFT) is used to transform the time series after Scrubbing (parameter: taper percentage = 0, FFT length = shortest), and then the power spectrum is obtained. Since the power of a given frequency is proportional to the square of the amplitude of this frequency component in the time domain of the original time series, the square root of the power spectrum is calculated at the given frequency range and the root mean square of 0.01∼0.08 Hz is obtained at each voxel, which is called ALFF. For standardization purposes, the ALFF of each voxel is divided by the global mean ALFF value.

### Statistic analysis

Demographic and clinical characteristics were evaluated among four groups. Two-sample *t*-tests or the Mann-Whitney *U*-test were used for the demographic characteristics (age and education level) and clinical scores (smoking years, pack year, and FTND score). Differences were considered significant at *P* < 0.05.

Using the full factorial model in SPM12, we conducted two-way analysis of variance (ANOVA) to analyze the two factors—smoking (smokers and non-smokers) and weight status (overweight and normal-weight)—of whole-brain ALFF maps (Yang et al., 2019). Education, age, and the mean FD were entered as covariates. The results were set at a threshold of *P* < 0.05 (voxel threshold *P* < 0.005 and cluster extent threshold *P* < 0.05, GRF corrected).

Each identified cluster where ALFF was found to be significant for the effect of both smoking and BMI was set as the region of interest (ROI). The ALFF value was extracted from the ROI, and then *post hoc* comparisons were performed using a two-sample *t*-test for interaction effect analyses (*P* < 0.0125, Bonferroni correction).

### Correlation analyses

To examine the association of ALFF change with smoking severity and BMI status, we performed Spearman’s correlation analyses between brain regions (altered in interaction effect analyses) and clinical data (smoking years, pack-year, FTND score, and BMI value).

## Result

### Demographics and clinical characteristics

Demographic and clinical characteristics were shown in Table 1. Smokers did not differ from non-smokers in age, education years and mean FD (two sample *t*-test, *t* = −0.027, *P* = 0.979; *t* = 0.449, *P* = 0.654; Mann-Whitney *U*-test, *Z* = −1.714, *P* = 0.086, respectively). Smokers and non-smokers who are overweight did not differ from normal weight people in age, education years and mean FD (details in Table 1). Smokers with high body weight did not differ from normal-weight controls who smoked in FTND (*t* = 0.297, *P* = 0.767), smoking years (*t* = 1.871, *P* = 0.066), and pack-years (*t* = 1.919, *P* = 0.060).

**TABLE 1:** Demographic and clinical characteristics.

Demographics	Smokers (*n* = 64)		Non-smokers (*n* = 46)		Comparison		
			
	OW-SM (*n* = 34)	NW-SM (*n* = 30)	OW-NSM (*n* = 22)	NW-NSM (*n* = 24)	SM vs. NSM	OW vs. NW	ANOVA
Sex (M/F)	34/0	30/0	22/0	24/0	−	−	−
Race	Mongoloid	Mongoloid	Mongoloid	Mongoloid	−	−	−
Age (years)	35.03 ± 6.37	33.03 ± 7.08	36.68 ± 7.36	28.50 ± 4.80	*t* = −0.027, *P* = 0.979	*t* = 1.969, *P* = 0.052	*F* = 2.170, *P* = 0.096
Education (years)	15.35 ± 0.98	14.97 ± 1.77	14.77 ± 2.77	16.67 ± 2.14	*Z* = −1.714, *P* = 0.086	*Z* = −1.326, *P* = 0.185	*F* = 2.027, *P* = 0.115
Mean FD	0.10 ± 0.08	0.11 ± 0.10	0.09 ± 0.06	0.09 ± 0.04	*t* = 0.449, *P* = 0.654	*t* = 0.487, *P* = 0.628	*F* = 2.063, *P* = 0.110
FTND	4.77 ± 1.78	4.63 ± 1.75	−	−	−	*t* = 0.297, *P* = 0.767	−
Pack-year	19.26 ± 10.04	13.95 ± 12.09	−	−	−	*t* = 1.919, *P* = 0.060	−
Smoking year	16.00 ± 5.43	13.07 ± 7.09	−	−	−	*t* = 1.871, *P* = 0.066	−
Weight (kg)	82.06 ± 8.39	68.99 ± 5.65	78.82 ± 7.64	66.5 ± 6.21	*t* = 1.070, *P* = 0.287	*t* = 10.737, *P* = 0.000	−
BMI	26.84 ± 2.07	21.88 ± 1.56	26.60 ± 1.98	22.11 ± 1.31	*t* = 0.443, *P* = 0.659	*t* = 14.059, *P* = 0.000	−

SM, smokers; NSM, non-smokers; OW, overweight; NW, normal weight; FTND, Fagerström Test for Nicotine Dependence; BMI, body mass index; M, males; F, females.

### The main effects

**FIGURE 1:**
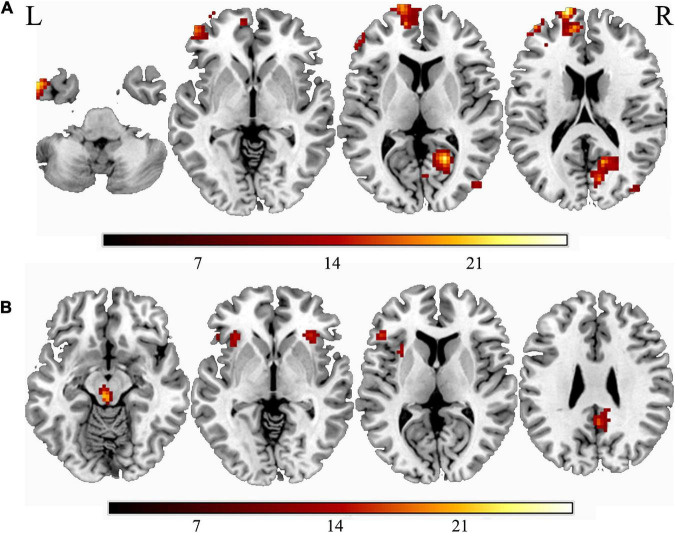
The main effect of smoking or weight. **(A)** The main effect of smoking; **(B)** the main effect of overweight. “L” means the side is to the left and “R” means the side is to the right.

The main effect of smoking was found in the left ITG, left SFG, middle frontal gyrus, right calcarine sulcus, right precuneus, and the right MTG. Compared with non-smokers, smokers group indicated increasing ALFF value in all brain regions mentioned above (Figure 1A and Table 2). The main effect of weight was found in the midbrain, left insula, bilateral inferior frontal gyrus, left SFG, and right precuneus (Figure 1B and Table 2), with overweight groups exhibiting elevated brain spontaneous fluctuations than normal-weight groups.

**TABLE 2:** Significant group differences in amplitude of low-frequency fluctuation.

Brain region	MNI coordinates (X, Y, Z)	Cluster size	Peak *F*-value
**Interaction effect**			
MTG_L	−63, −33, −12	33	21.780
**Main effect of smoking**			
ITG_L	−51, 6, −45	58	23.153
SFG_L MFG_L	−15, 69, 18	514	26.116
Calcarine_R/precuneus	21, −60, 12	319	27.643
MOG_R/MTG_R	45, −78, 12	56	15.621
**Main effect of overweight**			
Midbrain/brainstem_L	−3, −27, −9	44	22.185
Insula_L	−36, 18, 0	77	17.674
IFG_R	36, 24, 0	47	15.202
IFG_L	−45, 27, 6	34	15.276
SFG_L	−21, 48, 18	35	17.099
Precuneus_R	3, −51, 30	189	28.661

MTG, middle temporal gyrus; ITG, inferior temporal gyrus; SFG, superior frontal gyrus; MFG, middle frontal gyrus; MOG, middle occipital gyrus; IFG, inferior frontal gyrus; MSFG, medial superior frontal gyrus; L, left; R, right.

### Interaction effects

**FIGURE 2:**
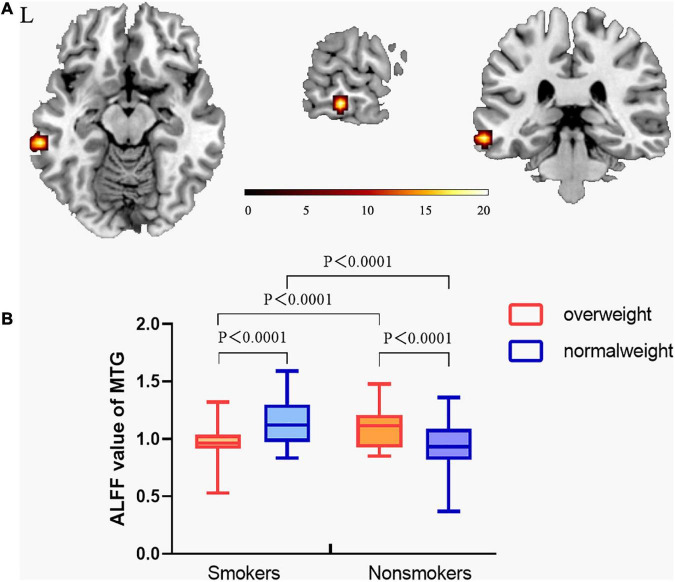
The interaction effect between smoking and overweight. **(A)** A significant interaction effect shown by ALFF in the MTG using two-way ANOVA. The statistical significance level was set at voxel threshold *P*< 0.005 and cluster extent threshold *P* < 0.05, GRF corrected. **(B)** *Post hoc* analysis of the MTG among the four groups. From this figure, normal-weight smokers had increased ALFF value in middle temporal gyrus compared with normal-weight non-smokers. However, the ALFF value of the middle temporal gyrus of overweight smokers was still higher than that of normal-weight non-smokers, but the difference decreased. This suggests that the ALFF value of the middle temporal gyrus of overweight smokers is closer to that of normal-weight non-smokers, compared to that of normal-weight smokers. (The vertical bar indicates the maximum and minimum across subjects. The bars in the box represent the mean ALFF for each group.) “L” means the side is to the left.

The interaction effect between smoking and weight was showed in the MTG (Figure 2A). *Post hoc* analysis showed normal-weight smokers had increased ALFF value in the MTG compared with normal-weight non-smokers (*t* = 3.554, *P* = 0.001). However, the mean ALFF values of the MTG were more similar between overweight smokers and normal-weight non-smokers (see details in Figure 2B).

### Correlation analysis

**FIGURE 3:**
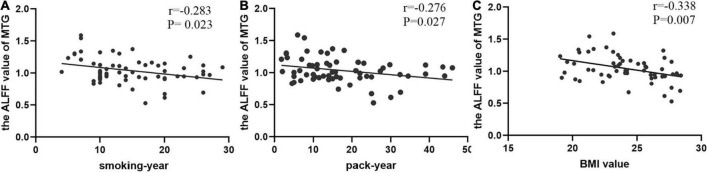
The correlations between the value of ALFF in the middle temporal gyrus (MTG) and clinical data. **(A)** In smokers, smoking-year was negatively correlated with the value of MTG (Spearman’s correlation, *r* = –0.283, *p* = 0.023); **(B)** pack-year was negatively correlated with the value of MTG (Spearman’s correlation, *r* = –0.276, *p* = 0.027); **(C)** BMI value was negatively correlated with the value of MTG (Spearman’s correlation, *r* = –0.338, *p* = 0.007).

Spearman’s correlation analysis found that the value of ALFF in the MTG was negatively correlated with smoking years, pack year, and BMI value (*r* = −0.283, *P* = 0.023; *r* = −0.276, *P* = 0.027; *r* = −0.338, *P* = 0.007, respectively) in smokers (Figure 3).

## Discussion

The current study used a 2 × 2 factorial design for smoking and weight status. We identified the spontaneous brain activity among four groups of subjects using ALFF method. Compared to non-smokers, smokers displayed raised brain activity in the left ITG, left SFG, middle frontal gyrus, right calcarine sulcus, right precuneus, right middle occipital gyrus, and right MTG no matter how much their weight. Moreover, overweight people had high ALFF value in the midbrain, left insula, bilateral inferior frontal gyrus, left SFG and right precuneus. More importantly, however, we found that smoking and body weight affected the spontaneous brain activity in the left MTG due to the interaction effect, which provides a possible explanation for their comorbidity.

The smoking main effect showing changed spontaneous fluctuations in the left ITG, left SFG, middle frontal gyrus, right calcarine sulcus, right precuneus, right middle occipital gyrus, and the right MTG, which was consistent with previous studies. Right calcarine sulcus, right middle occipital gyrus, and right MTG are involved in the formation of the visual cortex. Gallivan et al. (2011) found that a smoker’s attention is biased toward smoking cues, which potentially due to early processing changes in the visual system and reward-related areas. Changes in these brain regions in smokers may lead them to be more likely to associate smoking behavior with smoking cues in the environment when faced with temptation (such as watching other people smoking or pictures of cigarettes) (Gallivan et al., 2011). Left superior frontal gyrus (SFG), left ITG, and right precuneus are parts of the DMN. SFG is anatomically connected with the cingulate cortex (mostly the anterior cingulate and the middle cingulate, involved in cognitive control) through the cingulum and that it is functionally correlated with the default mode network (DMN) (Li et al., 2013). Abnormalities in the DMN can be observed in people with different substance use disorders (Zhang and Volkow, 2019). In short, failed inhibition of DMN activity during tasks in substance addiction was associated with increased drug-cue reactivity and impaired cognitive control (Garavan et al., 2000; Bednarski et al., 2011; Vollstädt-Klein et al., 2011). The cognitive impairment of addicts may be partly due to their impaired ability to relax emotional involvement in cognitive control processes and to shift attention from self-generated thoughts to external stimuli, mediated by interruptions in pre-DMN and post-DMN activity, respectively (Zhang and Volkow, 2019). These brain changes suggest when smokers faced with a temptation, have a bias of attention and are more likely to elicit the urge to smoke.

The main effect of overweight was found in the midbrain, insula, bilateral inferior frontal gyrus and right precuneus. Midbrain, a brain region composed largely of DA (60–65%), γ-amino butyric acid (GABA; ∼30–35%) and glutamatergic neurons (2–3%), is the core components of reward circuitry (Cooper et al., 2017). The feeding behavior is partly regulated by the mesolimbic dopamine system (Berridge and Robinson, 1998), and the neurotransmitter dopamine plays a crucial role in the regulation of food reward within this system. If the body lacks dopamine receptors, it becomes insensitive to excitement. For example, overweight people, who lack dopamine receptors, tend to receive food stimuli more slowly than normal people, so they need more food to satisfy their pleasure. The NAc represents the central to desire and is an important anatomical component of reward circuitry, receiving extensive projections of DA neurons from the ventral tegmental area (VTA) of the midbrain (Ikemoto and Wise, 2004). Activation of these midbrain DA neurons is associated with the motivating, rewarding and motivational saliency properties of natural stimuli such as food and drug abuse (Richardson and Gratton, 1996; Ikemoto and Wise, 2004; Hardman et al., 2012). Moreover, the neural mechanisms of food reward are thought to be similar to drug rewards. The insula was also modulated by dopaminergic activity to involve in motivation, rewards, and cognitive control (Chikama et al., 1997), which can enhance reward drive and weaken cognitive control. Therefore, altered insula activity may result in lower self-control ability and increased distractibility (Gao et al., 2021), this causes people to increase their eating behavior and gain weight. In addition, increased ALFF value in bilateral inferior frontal gyrus and right precuneus in our study, these brain regions are parts of a network involved in executive control function (ability to inhibit behavioral responses in the face of distractions) (Hardee et al., 2020). Prior studies have linked changes in this functional network, interpreted as less efficient processing, to risk for substance use problems (such as food) (Heitzeg et al., 2015).

In our research, the interaction effect of smoking and overweight status was observed in the MTG. Previous studies have found changes in MTG brain activity in both smokers (Xue et al., 2020) and overweight people (Hardee et al., 2020). MTG has a variety of functions including emotion, memory, and cognition (Rizzolatti et al., 1996; Giraud et al., 2004; Hesling et al., 2005; Sato et al., 2012), whose impairments have been reported to be associated with various brain disorders, such as major depression disorders (Cheng et al., 2019) and obsessive-compulsive disorder (Fan et al., 2017). Based on anatomical and functional connectivity, Xu et al. (2015, 2019) found bilateral anterior MTG (aMTG) mainly connected with left precuneus and SFG (two key nodes of the DMN). Fingerprint analysis further confirmed this, indicating a high functional connection between aMTG and DMN. Furthermore, during external-oriented tasks, drug-cues and food-cues elicited MTG activity in substance use disorder people, which perhaps is related with shifting attention from self-related thoughts to external stimuli (Andrews-Hanna et al., 2014; Tomasi et al., 2015). This suggests that when smokers see smoking cues, their desire to smoke trumps their inner desire not to smoke. Above failed inhibition of MTG and DMN activity during tasks in substance addiction was associated with increased drug-cue reactivity and impaired cognitive control (Bednarski et al., 2011; Zhang and Volkow, 2019). In addition, it has been reported that activity in the left posterior MTG increases as the number of suggestive movements associated with the object being observed increases (Schubotz et al., 2014). Recently, it has been found that posterior MTG is related to the proper objects grasping ability (Amoruso et al., 2018). All evidence suggested that the posterior MTG might play an important role in action observation and integrating the motoric details of the action. Overall, our findings show the MTG plays an integrated role in motor observation, external information response and cognitive control, these functions in the MTG provide a possible neurological explanation for the altered MTG in both overweight and smokers, and may be one of the possible reasons for their frequent comorbidities.

Additionally, *Post hoc* analysis of the MTG showed that normal-weight smokers have increased ALFF value with regard to normal-weight non-smokers, which is consistent with previous research (Gearhardt et al., 2014). However, the ALFF value of the MTG of overweight smokers is closer to that of normal-weight non-smokers, compared to that of normal-weight smokers. In a word, smoking activates spontaneous brain fluctuations in the MTG, and overweight mitigated these changes in smoking populations, this suggests that smoking and being overweight have opposite effects on the activity of the MTG. In all smokers, the value of ALFF in the MTG was negatively correlated with smoking years, pack year and BMI value (*r* = −0.283, *P* = 0.023; *r* = −0.276, *P* = 0.027; *r* = −0.338, *P* = 0.007, respectively).

There are several limitations to consider when designing our study. Firstly, all subjects were males, which is because (1) the smoking rate of males is much higher than that of females, the issue of male smoking needs to be focused on (Nakagawa et al., 2022); (2) It has been reported that gender has an effect on the brain function of smokers (Zhang et al., 2017), therefore, in order to control the influencing factors, only males were included in our study. Secondly, the overweight volunteers and smokers we included lacked a few clinical data (e.g., abdominal circumference and serum cholesterol level in overweight people; exhaled carbon monoxide levels in smokers), so we couldn’t conduct correlation analysis between the different brain regions with these data. Thirdly, this study included daily smokers with low cigarette count. Because even if a smoker may smoke one cigarette a day, he meets the criteria for nicotine addiction, so we count him as a smoker (Wen et al., 2021). At the same time, studies have found that daily smokers may have higher mortality rates (Inoue-Choi et al., 2019) and lower awareness of clinical prevention services (Vander Weg et al., 2012) than non-daily smokers, so we feel it is important to highlight the concept of daily smoking. Fourthly, this study excluded obese population by limiting BMI to 30. Studies have shown that overweight differs substantially from obesity. A study of the effect of BMI on intra-network rest-state connectivity in smokers showed heavier smoking was related to greater rsFC in the salience network among lean and obese groups but reduced rsFC in the overweight group (Ely et al., 2021). At the same time, BMI had a significant secondary effect on DMN connectivity. In addition, Luo et al. (2018) showed that the voxel-mirrored homotopic connectivity (VMHC) in the calcarine gyrus and post-central gyrus was different in obese and overweight individuals. In a word, focusing on overweight is a separate issue and should be treated as such. Finally, this study was cross-sectional and the sample size of this study is relatively small, so more data can be collected for analysis in future studies, making the statistical effect higher and the conclusion more reliable.

## Conclusion

In this study, we found that both smoking and body weight affect spontaneous brain activity, and a novel antagonistic interaction effect of smoking and overweight in the MTG of smokers who are overweight. This suggests that we need to control for weight as a variable when studying spontaneous brain activity in smoking. In addition, the interaction between smoking and overweight on the MTG may provide a neurological explanation for the comorbidities between overweight and smoking and a target for treatment of the comorbidities.

## Data availability statement

The raw data supporting the conclusions of this article will be made available by the authors, without undue reservation.

## Ethics statement

The studies involving human participants were reviewed and approved by the Ethics Committee of the First Affiliated Hospital of Zhengzhou University. The patients/participants provided their written informed consent to participate in this study.

## Author contributions

XG: data curation, formal analysis, methodology, visualization, and writing—original draft. MZ: data curation, formal analysis, methodology, and writing—original draft. ZY and SH: methodology and writing—review and editing. XN and JCn: methodology. BZ and YW: writing—review and editing. WW: resources. YZ: conceptualization, funding acquisition, methodology, resources, supervision, and writing—review and editing. JCg: conceptualization, funding acquisition, resources, supervision, and writing—review and editing. All authors contributed to the article and approved the submitted version.

## Footnotes

1
www.restfmri.net
